# Association between Drug Insurance Cost Sharing Strategies and Outcomes in Patients with Chronic Diseases: A Systematic Review

**DOI:** 10.1371/journal.pone.0089168

**Published:** 2014-03-25

**Authors:** Bikaramjit S. Mann, Lianne Barnieh, Karen Tang, David J. T. Campbell, Fiona Clement, Brenda Hemmelgarn, Marcello Tonelli, Diane Lorenzetti, Braden J. Manns

**Affiliations:** 1 Department of Medicine, University of Calgary, Calgary, Alberta, Canada; 2 Interdisciplinary Chronic Disease Collaboration Team, Calgary, Alberta, Canada; 3 Department of Community Health Sciences, University of Calgary, Calgary, Alberta, Canada; 4 Institute of Public Health, University of Calgary, Calgary, Alberta, Canada; 5 Libin Cardiovascular Institute of Alberta, University of Calgary, Calgary, Alberta, Canada; 6 Department of Medicine, University of Alberta, Edmonton, Alberta, Canada; National Institute for Public Health and the Environment, Netherlands

## Abstract

**Background:**

Prescription drugs are used in people with hypertension, diabetes, and cardiovascular disease to manage their illness. Patient cost sharing strategies such as copayments and deductibles are often employed to lower expenditures for prescription drug insurance plans, but the impact on health outcomes in these patients is unclear.

**Objective:**

To determine the association between drug insurance and patient cost sharing strategies on medication adherence, clinical and economic outcomes in those with chronic diseases (defined herein as diabetes, hypertension, hypercholesterolemia, coronary artery disease, and cerebrovascular disease).

**Methods:**

Studies were included if they examined various cost sharing strategies including copayments, coinsurance, fixed copayments, deductibles and maximum out-of-pocket expenditures. Value-based insurance design and reference based pricing studies were excluded. Two reviewers independently identified original intervention studies (randomized controlled trials, interrupted time series, and controlled before-after designs). MEDLINE, EMBASE, Cochrane Library, CINAHL, and relevant reference lists were searched until March 2013. Two reviewers independently assessed studies for inclusion, quality, and extracted data. Eleven studies, assessing the impact of seven policy changes, were included: 2 separate reports of one randomized controlled trial, 4 interrupted time series, and 5 controlled before-after studies.

**Findings:**

Outcomes included medication adherence, clinical events (myocardial infarction, stroke, death), quality of life, healthcare utilization, or cost. The heterogeneity among the studies precluded meta-analysis. Few studies reported the impact of cost sharing strategies on mortality, clinical and economic outcomes. The association between patient copayments and medication adherence varied across studies, ranging from no difference to significantly lower adherence, depending on the amount of the copayment.

**Conclusion:**

Lowering cost sharing in patients with chronic diseases may improve adherence, but the impact on clinical and economic outcomes is uncertain.

## Introduction

Access and adherence to medications is important in the management of many chronic diseases, including cardiovascular conditions [1]–[5]. Since medications are a major driver of health expenditures [6], insurance plans have instituted a variety of cost sharing strategies, such as copayments, aimed at reducing expenditure on pharmaceuticals [7] (Box 1).

The type of drug insurance that is available to citizens varies internationally, as does the use of patient cost sharing strategies. Many patients lack drug insurance or make some form of direct payment for a portion of their prescriptions, which may constitute a financial barrier to drug access [8], [9] – especially since patients with lower socioeconomic status are at higher risk of chronic diseases [10]–[12]. In a recent survey of Canadians with hypertension, diabetes or cardiovascular disease, nearly 10% identified a financial barrier to accessing drugs, and those with barriers were 50% less likely to receive statins than those without barriers [13].

A prior Cochrane review of studies published before 2008 found low-quality evidence that fixed copayments and caps reduced adherence to medications [14]. A separate review found that higher levels of copayments were associated with poor adherence, discontinuation and non-initiation of therapy [15]. We sought to update previous reviews and determine the impact of drug insurance (vs. no drug insurance) and varying levels of patient cost sharing (i.e. copayment, deductible, caps, and maximum out-of-pocket expenditure) on medication adherence, clinical and economic outcomes in patients with cardiovascular-related chronic disease. This work focused on cardiovascular related chronic diseases given that long-term medication use (in addition to lifestyle changes) is the mainstay of treatment in these conditions, and that a large body of evidence shows that selected preventative medications (i.e. antihypertensive agents, statins, and anti-diabetic drugs) are effective in reducing morbidity and mortality [16]–[19].

**Box 1. Definition of Cost-Sharing Strategies to Restrict Expenditures**

- *Full drug insurance:* a policy where the patient does not pay any out-of-pocket expenditure at the time the prescription is dispensed.
- *Cap:* a limit below which a patient does not pay or has reduced payments for prescriptions. After the cap is reached full payment is required by the patient.
- *Coinsurance:* a system where a patient pays a set percentage of the amount per drug or per prescription.
- *Copayment:* an amount per drug or per prescription that a patient pays.
- *Coverage gap:* a gap between a cap and catastrophic coverage where a patient is responsible for the full cost of the drug.
- *Deductible:* a limit up to which a patient pays the full cost of the drug. After the deductible is reached, the patient either does not pay or has reduced payments for prescriptions.
- *Fixed copayment:* a system where a patient pays a fixed, or set, amount per drug or per prescription.

*Maximum out-of-pocket limit:* a limit that is set as a fixed dollar amount or as a percentage of income after which the insurer pays 100% of the drugs. Copayments and coinsurance are in place prior to the limit being reached. Some studies refer to this as catastrophic coverage limit

## Methods

### Data Sources and Searches

A librarian assisted search was performed of electronic databases for English language studies, from inception until March 2013 and included: MEDLINE, EMBASE, CINAHL, Cochrane Controlled Trials Register, and Current Controlled Trials. The full search strategy is available in Appendix S1 – in brief, the key terms included: *coronary artery disease*, *hypertension*, *dyslipidemia*, *diabetes*, *peripheral vascular disease*, *stroke*, *transient ischemic attack*, *heart failure*, *chronic kidney disease*, *insurance*, *pharmaceutical services*, *health or for-profit insurance plans*, *reimbursement*, *Medicare*, *single-payer system*, *copay*, *deductibles*, *coinsurance*, and *insurance coverage*. No limitations were placed on patient characteristics, study duration or outcomes; the bibliographies of included studies were also searched independently by two reviewers (BSM and LB).

### Study Selection

Two reviewers (BSM and LB) independently screened citations and determined eligibility in two stages. In the first stage, all identified citations were reviewed, while the second stage encompassed full-text review of selected abstracts to determine eligibility. Disagreements were resolved by consensus or through consultation with a third reviewer (BJM). Studies were included if they focused on: adult patients with chronic disease (coronary artery disease, hypertension, diabetes, hypercholesterolemia, and cerebrovascular disease), and assessed the impact of full drug insurance without cost sharing, or with lower level of cost sharing as part of a drug insurance system against a comparator group.

We included studies that examined various cost sharing strategies including copayments, coinsurance, fixed copayments, deductibles and maximum out-of-pocket expenditures, defined in Box 1. The cost sharing strategy for the intervention group was the strategy with lower out of pocket payments for the patient, ranging from no payment at all (full drug insurance) to some form of payment. The comparator group had higher out of pocket payments for the patient and ranged from no drug insurance (full payment of pharmaceutical) to a higher level of payment relative to the intervention group through the use of cost sharing strategies such as copayments, fixed copayments, deductibles, coinsurance, or maximum out of pocket expenditures.

Consistent with the Cochrane Effective Practice and Organisation of Care Group [EPOC] taxonomy of health care policy studies [20], we included: randomized controlled trials (RCTs), non-randomized controlled trials, controlled before-after (CBA) and interrupted time series (ITS) studies. Relevant outcomes included: medication adherence, clinical events (myocardial infarction, stroke, death), quality of life, healthcare utilization, or cost. Studies were excluded if they: focused exclusively on children or adolescents, or patients with medical conditions other than chronic cardiovascular disease or one of its risk factors. Studies were further excluded if the health policy focus was value-based insurance, or reference based pricing.

### Data Extraction and Quality Assessment

Two reviewers (BSM and LB) independently extracted data and disagreements were resolved by consensus or through consultation with a third reviewer (BJM). The quality of included studies was evaluated using the Cochrane risk of bias tool for randomized controlled trials [21], as well as the Cochrane EPOC taxonomy for non-randomized trials, controlled before-after studies and interrupted time series designs [20].

### Data Synthesis and Analysis

As we anticipated substantial heterogeneity between the outcomes reported across studies, we developed broad categories of outcomes, and decided *a priori* not to pool the studies. Individual study results are reported by type of intervention.

## Results

**Figure 1:**
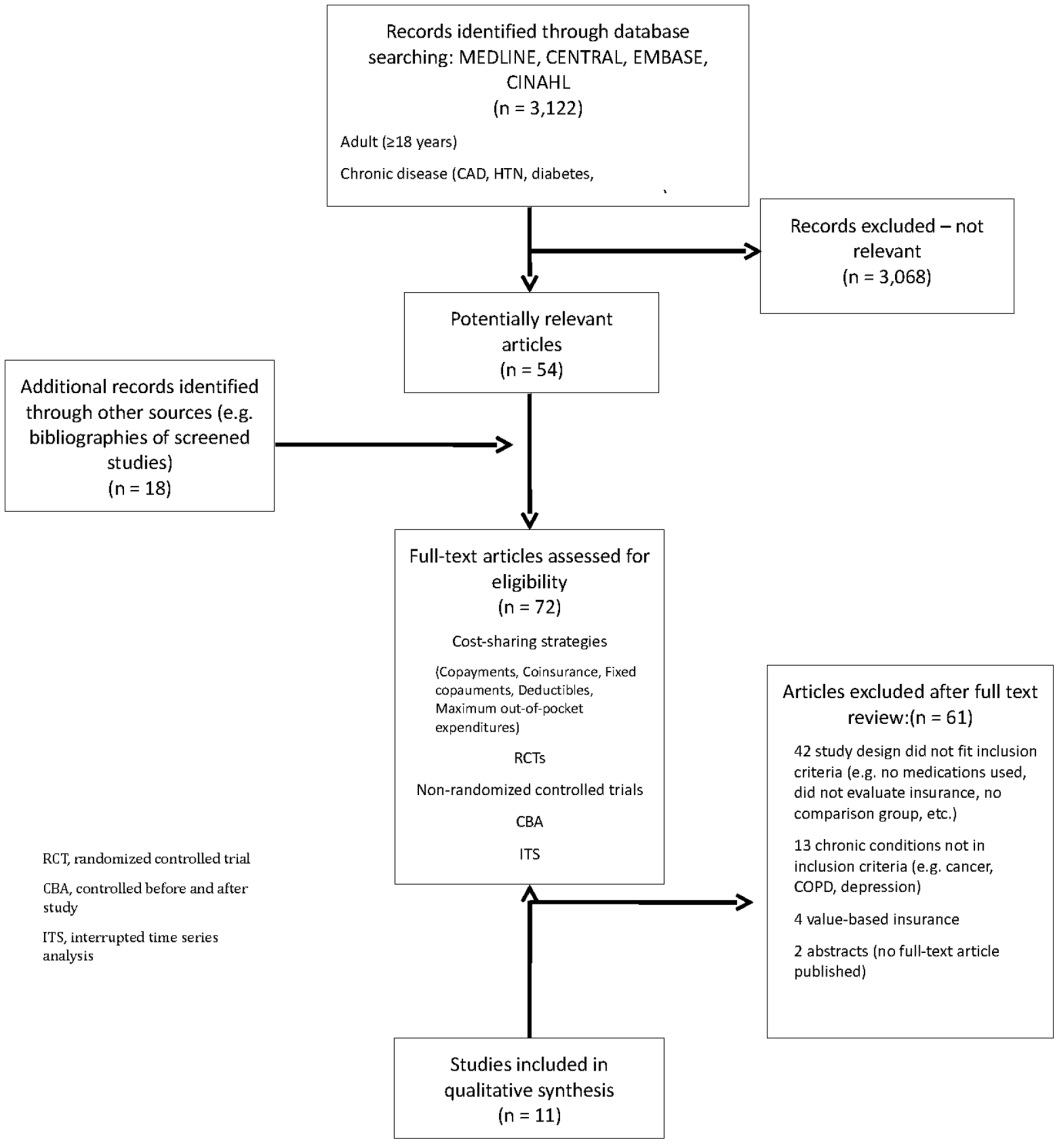
Flow diagram depicting included and excluded studies for the qualitative systematic review.

**Figure 2:**
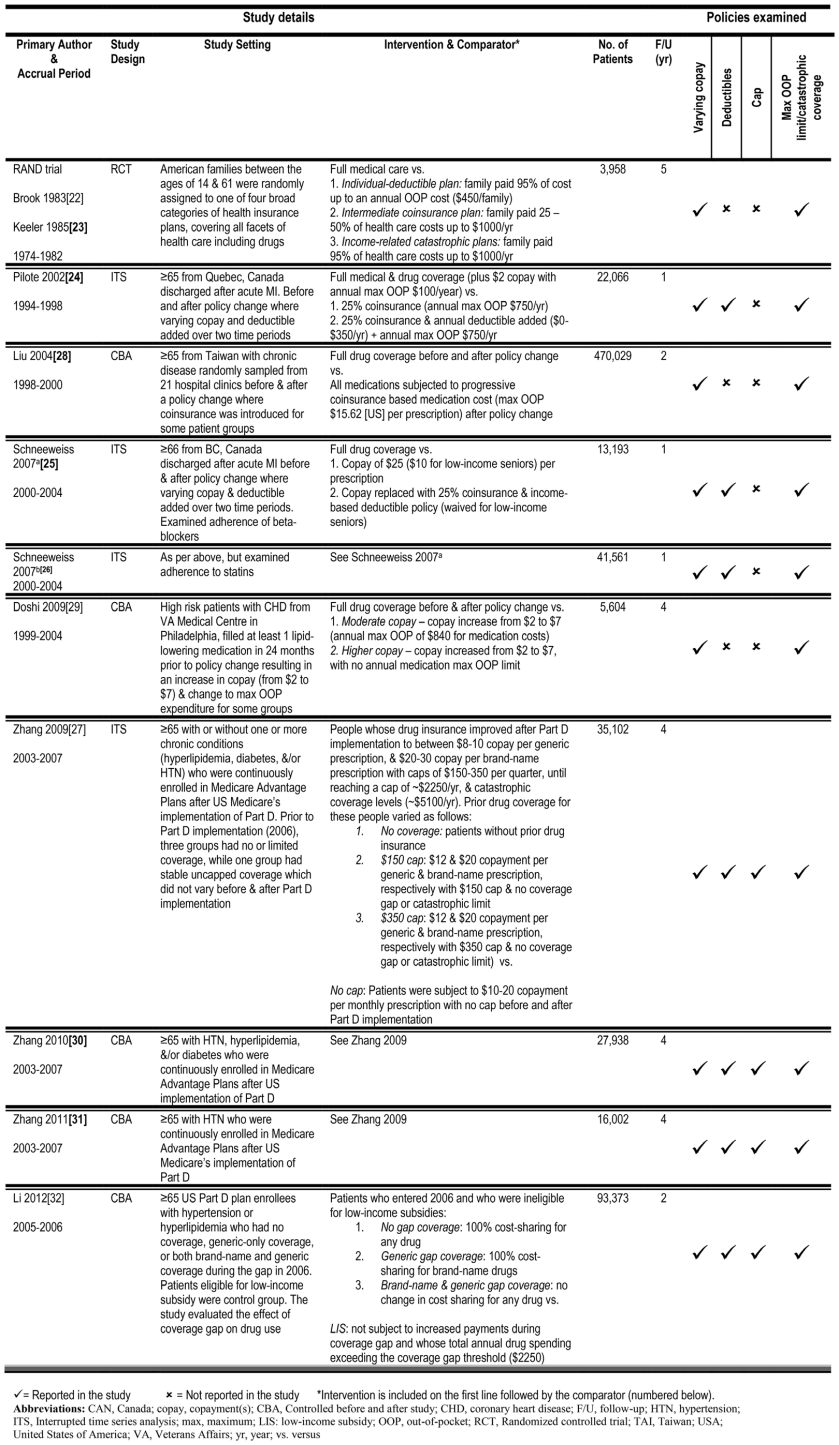
Overview of included studies.

The search yielded 3,122 citations, 72 of which were selected for full-text review. Of these, 11 studies evaluating 7 different drug insurance policy changes met our inclusion criteria (Figure 1): 2 separate reports of one randomized controlled trial (RCT), the RAND trial [22], [23]; 4 interrupted time series (ITS) assessing three drug policy changes [24]–[27]; and 5 controlled before-after (CBA) studies assessing three drug policy changes [28]–[32] (Figure 2). Seven of the studies were from the US, three were from Canada, and one was from Taiwan with eight of the eleven studies focusing on elderly patients.

### Quality of Included Studies

The randomized controlled trial was rated as moderate quality (Appendix S2). The other studies included in our review consisted of ITS designs (rated as high quality) and CBA studies (rated as relatively poor quality) (Appendix S2).

### Having Drug Insurance, Compared with No Insurance

**Figure 3:**
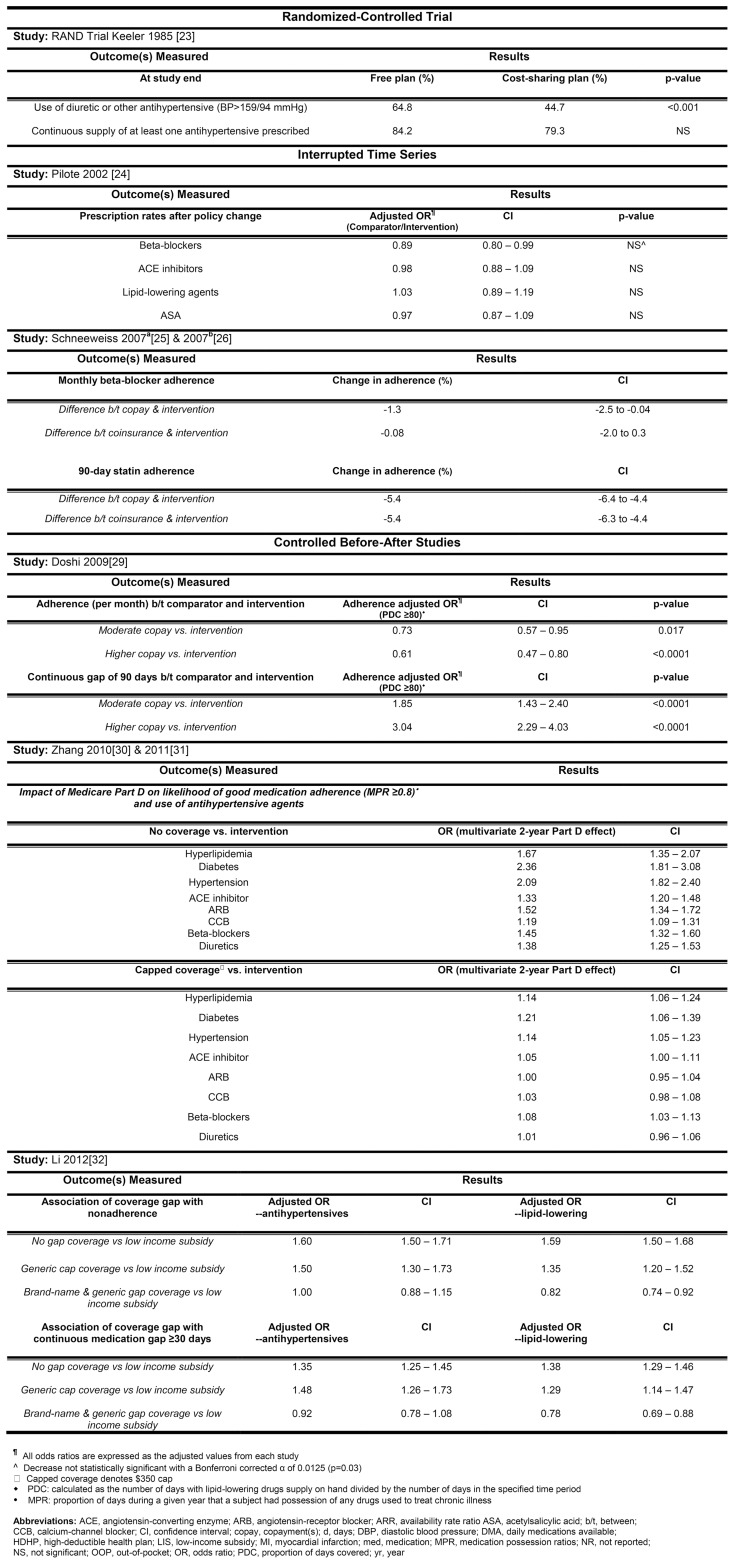
The association between cost sharing and medication utilization and adherence.

Three studies compared those with no drug coverage to a group that had stable uncapped drug coverage that remained unchanged before and after the implementation of Medicare Part D [27], [30], [31]. They found that in patients aged 65 or older with hypertension, hypercholesterolemia, and/or diabetes, drug insurance increased the odds of adherence to guideline-recommended medications by 19–136% compared to those without drug insurance coverage (Figure 3). In hypertensive patients aged 65 or older, drug insurance was associated with a 2-fold increase in the odds of using an antihypertensive agent compared to those without drug insurance (Figure 3).

**Figure 4:**
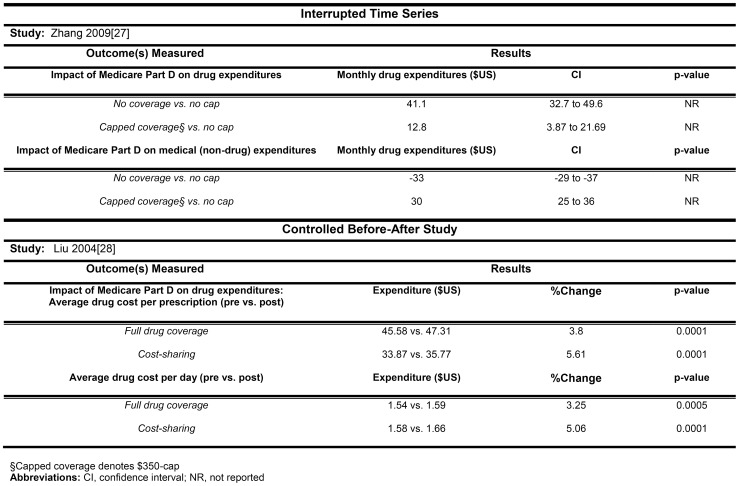
The association between cost sharing and drug and non-drug expenditures.

Zhang 2009 [27] also examined drug expenditures and found that compared to those without drug insurance, drug insurance as offered by Medicare Part D coverage was associated with higher drug expenditures ($41 per month), but lower nondrug health care expenditures for each patient (Figure 4). None of these studies evaluated clinical outcomes.

### Different Levels of Copayment

Seven studies (assessing five different policy interventions) assessed the impact of varying levels of copayment, ranging from full coverage (no payment from patient) to 95% patient copayment on drug adherence (Figure 3).

The RAND RCT compared full healthcare insurance with three different cost sharing strategies. In a sub-group analysis, they found that in patients with hypertension, full drug insurance led to higher use of antihypertensive medications at the exit screening examination (20% absolute increase) compared to each of the three cost sharing strategies [23]. One study from Quebec found no apparent decrease in medication adherence (Figure 3) when a minimal copayment was changed to 25% coinsurance [24] (see Figure 2 for more detail on policy change). Another study from British Columbia noted no change in beta-blocker initiation in elderly post-myocardial infarction patients when full drug coverage was changed to a fixed copayment, with a subsequent addition of coinsurance plus deductible. However, a small decrease was noted in adherence over time [25]. A third study examined the association between the same policy change and use of statins in British Columbia, and found no decrease in statin initiation over time, but did observe a 5% decrease in adherence during follow-up [26]. In a high risk group of US veterans with coronary disease [29], increasing copayment by $5 per prescription, with or without an annual maximum out-of-pocket expenditure, resulted in a 30–40% lower adjusted odds of adherence across a variety of measures (Figure 3).

**Figure 5:**
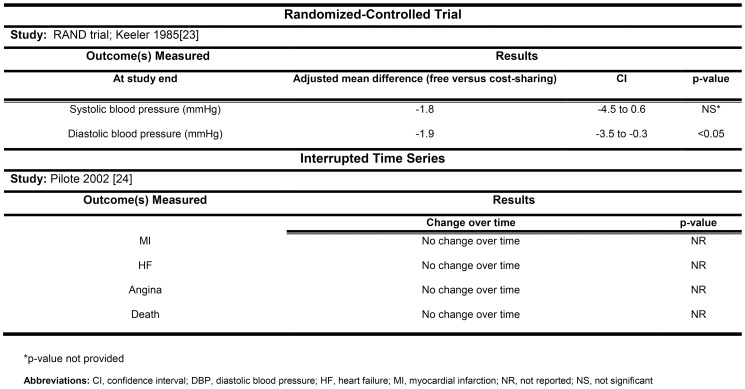
The association between cost sharing and clinically relevant outcomes.

Clinically relevant outcomes were reported in two studies. The sub-group analysis of the RAND trial reported that full insurance was associated with a statistically significant decrease in diastolic blood pressure (−1.9 mmHg, 95% CI −3.5 to −0.3 mmHg), as compared to the three cost sharing strategies (Figure 5). The Quebec study found no difference in death, myocardial infarctions, heart failure, or angina after changing from a small copayment to 25% coinsurance (Figure 5). Though expenditures were not reported in any of the studies, the addition of a 25% copayment did not appear to increase admissions to hospital or the emergency room in Quebec [24].

### Deductibles

The impact of deductibles was assessed in the studies from Quebec and British Columbia [24]–[26]. The Quebec study found that introduction of the cost sharing strategy was not associated with a decrease in drug use (Figure 3). In the two British Columbia studies, evaluating one policy, there was no association between introduction of the deductible and adherence to beta-blockers or statins, compared with only a fixed copayment. However, being subject to a 100% copay, for those who had not yet reached their deductible, was associated with a 2-fold increase in the risk of discontinuing statins [26].

### Maximum Out-of-Pocket Limits and Coverage Gaps

Few studies evaluated changes in the maximum out-of-pocket limit, outside of other policy changes. In a high risk group of US Veteran's Administration patients with coronary heart disease, there was a slight decline in adherence in patients without an annual maximum out-of-pocket expenditure, compared to those with a maximum out-of-pocket expenditure ($840 US per year) (Figure 3) [29]. In Quebec, a change from minimal copayment ($2 CDN per prescription; annual maximum of $100 CDN) to 25% coinsurance, with a $250-$750 CDN annual maximum out-of-pocket expenditure had no apparent change in medication use (Figure 3).

A study by Li et al examined the effect of a coverage gap in a group of elderly Part D enrollees who were ineligible for low-income subsidies with elderly Part D enrollees who were eligible for low-income subsidies. As of 2006, the standard Medicare Part D design has a coverage gap, where a beneficiary has to pay 100% of drug costs after total drug spending exceeds an annual threshold of $2250 in 2006 [32]. This study found that the Part D coverage gap was associated with a 1.5-fold increased probability in nonadherence to antihypertensive and lipid-lowering agents in those who entered the coverage gap phase in 2006 with no coverage (Figure 3).

## Discussion

Our systematic review identified 11 studies, comparing 7 policy changes assessing drug insurance and patient cost sharing strategies (i.e. copayment, deductible, caps, and maximum out-of-pocket expenditure) in patients with or at risk for cardiovascular disease. Although data on clinical outcomes was not widely reported, our review found that providing drug insurance to people with chronic diseases who have no drug insurance appears to increase appropriate use of and adherence to drugs.

The proportion of the US gross domestic product that is spent on health care has more than tripled over the past 30 years [33]. Prescription drugs constitute a significant proportion of overall healthcare costs, with current annual spending for prescription drugs in the US at $259 billion, projected to double over the next decade [34]. One mechanism that has been devised to reduce the financial burden to insurance plans is to shift the burden from the insurer to patients [35], [36]. This shift of financial responsibility may lead to underuse of potentially important medications in people with chronic conditions [37], [38].

Previous reviews have examined the relationship between drug insurance and health care utilization [39] or various policies regarding direct payment of drugs [14]. Our search strategy, and the results of our search, were compared with the Cochrane review [14]. Our search strategy captured all relevant studies identified in the Cochrane review, along with additional newer studies which focused on the subset of people with or at risk for cardiovascular disease. Like others, we found that the addition of drug insurance for those without previous drug insurance appears to consistently increase adherence to medications, and that increased costs on drug expenditures may be offset by decreased costs in non-drug expenditures [27], [30], [31]. In general, studies evaluating cost sharing strategies appeared to have conflicting results with some studies showing significant differences in some outcomes such as the RAND trial [23], Doshi [29], and Zhang 2010 and 2011 [30], [31]; while other studies such as the studies out of Quebec [24] and British Columbia [25], [26] demonstrated no discernable difference in outcomes. Since the cost sharing policies differed from study to study it is worthwhile discussing the different strategies used in the studies in our review. In the studies we included, the use of increasing patient copayments (up to 25% patient copayment) does not appear to reduce the appropriate initiation of medications, but is associated with a small reduction in medication adherence, when compared to full drug coverage. The RAND trial had a significant increase in copayments, which included up to 95% copayments as part of its cost sharing strategy. This study also provided insights into the change in adherence associated with full drug insurance, as it noted a 20% absolute increase to antihypertensive agents compared to the cost sharing group. As such, we can infer that a small copayment (up to 25%) does not appear to impact adherence, while large copayments (95% copay) may have a substantial impact on medication adherence. Furthermore, the impact on adherence may be more significant in those with low socioeconomic status [29], providing some insight into vulnerable populations in whom policy makers may consider waiving copayments.

While the use of deductibles (up to $350 per year) does not appear to have a significant impact on medication adherence, one study reported that 100% copayment (i.e. those who had not yet reached the deductible level) was associated with a two-fold reduction in drug adherence [26]. Furthermore, when patients exceed a pre-defined annual threshold limit and enter a period of a coverage gap the use of medications decreases, particularly when patients were responsible for 100% of medication costs compared to those who had some form of drug coverage [32]. Waiving deductibles and the coverage gap for those from a lower socioeconomic status may be a consideration for policy makers. It is uncertain whether there is a linear relationship between the deductible and adherence though our results suggest there may be a threshold effect. Finally, the impact of a maximum out-of-pocket limits was uncertain [24], [29].

Our review has some limitations: we limited our search to English language studies and may have missed non-English language studies. Furthermore, the heterogeneity of the studies prevented us from obtained pooled estimates of the overall effect of drug insurance on our outcomes of interest. Our review focused on patients with or at risk for cardiovascular diseases, and it is possible that the impact of some tools (such as maximum out-of-pocket limits) may be more important for patients receiving very high-cost drugs, such as those with cancer. While we attempted to assess the association between changes in drug policy and clinically relevant outcomes, studies rarely reported on these outcomes. Though we wished to explore any unwanted side effects of using less drugs as a result of these cost-sharing strategies, the studies included did not report specifically on this outcome. However, providing medication coverage appeared to improve medication adherence, though studies either did not find or did not report changes in clinically relevant outcomes such as myocardial infarction, heart failure, angina or death [24].

Our review shows that providing drug insurance to those with or at risk for cardiovascular disease who have no insurance improves drug adherence. The impact of cost sharing strategies is less certain, though patient cost sharing in people of lower socioeconomic status may adversely impact adherence. Policy makers should be aware that copayments and deductibles, while reducing cost for the payer, may influence medication adherence and ultimately health outcomes – especially for those of lower socioeconomic status.

## Supporting Information

Appendix S1 Search strategy.
(DOCX)

Appendix S2 Risk of bias summary table.
(DOCX)
