# Supplementary material for: Association between Drug Insurance Cost Sharing Strategies and Outcomes in Patients with Chronic Diseases: A Systematic Review
Source: PLoS One. 2014 Mar 25;9(3):e89168. doi: 10.1371/journal.pone.0089168 (PMC3965394; doi:10.1371/journal.pone.0089168)
Supplement: Appendix S1 — Search strategy. (DOCX) [file pone.0089168.s001.docx]

**Appendix 1 – Search strategy**

1. exp Kidney Diseases/

2. exp Hypertension/

3. exp Cardiovascular Diseases/

4. exp Stroke/

5. exp Diabetes Mellitus/

6. exp Respiratory Tract Diseases/

7. exp Obesity/

8. exp Neoplasms/

9. exp Mental Disorders/

10. (kidney disease* or kidney disorder* or renal insufficienc* or renal disorder* or dialysis or hypertensi* or heart disease or heart patient* or cardiovascular disease or cardiovascular disorder* or myocardial infarction* or heart attack* or (chronic adj10 disease*) or stroke or respiratory disease* or respiratory disorder* or asthma* or obesity or obese or cancer or depression or psychiatric or mental disorder*).tw.

11. 1 or 2 or 3 or 4 or 5 or 6 or 7 or 8 or 9 or 10

12. exp *Arthritis/

13. exp *HIV Infections/

14. exp *"Tobacco Use Cessation"/

15. exp *Inflammatory Bowel Diseases/

16. exp *Autoimmune Diseases/

17. (arthritis or arthritic or osteoarthritis or HIV or AIDS or inflammatory bowel* or crohn* or colitis or autoimmune).ti.

18. 12 or 13 or 14 or 15 or 16 or 17

19. 11 not 18

20. limit 19 to (english or french)

21. limit 20 to animals

22. limit 20 to (animals and humans)

23. 21 not 22

24. 20 not 23

25. limit 24 to (case reports or comment or editorial or letter)

26. 24 not 25

27. exp "Cost Sharing"/

28. Insurance, Pharmaceutical Services/

29. exp Fees, Pharmaceutical/

30. exp Insurance Coverage/

31. insurance, health/ or for-profit insurance plans/ or health benefit plans, employee/ or insurance, health, reimbursement/ or exp insurance, pharmaceutical services/ or medicare/ or single-payer system/

32. Insurance, Health, Reimbursement/

33. "Deductibles and Coinsurance"/

34. ((co-pay* or copay* or coverage or insurance or insuring or cap or coinsur* or co-insur* or reimburs* or plan or plans or fees or "cost share" or "cost sharing" or insurance or insuring) adj10 (drug or drugs or medication* or pharmaceutical*)).tw.

35. 27 or 28 or 29 or 30 or 31 or 32 or 33 or 34

36. 26 and 35

37. limit 36 to (clinical trial or controlled clinical trial or comparative study or meta analysis or randomized controlled trial)

38. (random* or trial or trials or placebo* or groups).tw.

39. Time Factors/ or cohort studies/

40. time series.tw.

41. (controlled adj3 before adj3 after).tw.

42. 38 or 39 or 40 or 41

43. 36 and 42

44. 37 or 43
