# Supplementary material for: Association between Drug Insurance Cost Sharing Strategies and Outcomes in Patients with Chronic Diseases: A Systematic Review
Source: PLoS One. 2014 Mar 25;9(3):e89168. doi: 10.1371/journal.pone.0089168 (PMC3965394; doi:10.1371/journal.pone.0089168)
Supplement: Appendix S2 — Risk of bias summary table. (DOCX) [file pone.0089168.s002.docx]

**Appendix 2 - Risk of bias summary table**

|  | Allocation sequence adequately generated? | Allocation Concealment? | Baseline characteristics similar? | Incomplete outcome data adequately addressed? | Blinded assessment of primary outcome? | Complete outcome reporting? | Was the study adequately protected against contamination? |
| --- | --- | --- | --- | --- | --- | --- | --- |
| **Randomized Controlled Trials** | | | | | | | |
| Brook 1983[^18^](#_ENREF_18) |  |  |  |  |  |  |  |
| Keeler 1985[^19^](#_ENREF_19) |  |  |  |  |  |  |  |
| **Controlled Before and After Studies** | | | | | | | |
| Liu 2004[^24^](#_ENREF_24) |  |  |  |  |  |  |  |
| Doshi 2009[^25^](#_ENREF_25) |  |  |  |  |  |  |  |
| Zhang 2010[^26^](#_ENREF_26) |  |  |  |  |  |  |  |
| Zhang 2011[^27^](#_ENREF_27) |  |  |  |  |  |  |  |
| Li 2012[^28^](#_ENREF_28) |  |  |  |  |  |  |  |

|  | Intervention independent of other changes? | | Shape of intervention pre-specified? | | Intervention unlikely to affect data collection? | Knowledge of intervention adequately protected? | | Incomplete outcome data adequately addressed? | | Complete outcome reporting? | Was the study adequately protected against contamination? | |
| --- | --- | --- | --- | --- | --- | --- | --- | --- | --- | --- | --- | --- |
| **Interrupted Time Series** | | | | | | | | | | | | |
| Pilote 2002[^20^](#_ENREF_20) | |  |  |  | | |  | |  |  | |  |
| Schneeweiss 2007^a^[^21^](#_ENREF_21) | |  |  |  | | |  | |  |  | |  |
| Schneeweiss 2007^b^[^22^](#_ENREF_22) | |  |  |  | | |  | |  |  | |  |
| Zhang 2009[^23^](#_ENREF_23) | |  |  |  | | |  | |  |  | |  |

Red= high risk; yellow = medium risk; green = low risk; blank = not rep
